# Misplaced links in the chain of survival due to an incorrect manual for the emergency call at public facilities

**DOI:** 10.1186/1865-1380-6-33

**Published:** 2013-09-04

**Authors:** Yutaka Takei, Taiki Nishi, Keiko Takase, Takahisa Kamikura, Hideo Inaba

**Affiliations:** 1 Department of Medical Science and Technology, Hiroshima International University, 555-36 Kurose-gakuendai, Higashi-hiroshima-shi, Hiroshima 739-2695, Japan; 2 Department of Emergency Medical Science, Kanazawa University Graduate School of Medicine, 13-1 Takara-machi, Kanazawa 920-8641, Japan; 3 Departments of Emergency Medical Science and Emergency Medical Center, Kanazawa University Graduate School of Medicine, 13-1 Takara-machi, Kanazawa 920-8641, Japan

**Keywords:** Out-of-hospital cardiac arrest, Emergency call, Public facilities, Questionnaire survey, Prospective cohort study

## Abstract

**Background:**

The incidence of delayed emergency calls and the outcome of out-of-hospital cardiac arrest (OHCA) may differ among public facilities when emergency calls are placed by institutional staff. The purpose of this study was to identify the actions prescribed in the rules and/or manuals of public facilities and to clarify whether the incidence of delayed emergency call placement and the outcome of OHCA differ among these facilities.

**Methods:**

We performed a questionnaire-based survey regarding emergency calls in public facilities in our community and analyzed our regional Utstein-based OHCA database.

**Results:**

Our questionnaire survey disclosed that the most common actions prescribed in the manuals or rules applied in care facilities and educational institutions are to report the situation when a cardiac arrest occurs and to follow the directions of a custodian or supervisor. The international web search disclosed that these actions are rarely prescribed in medical emergency manuals in other countries. Most of these manuals simply say that staff should make an emergency call immediately upon detecting a serious illness or medical emergency. Analysis of the Utstein-based database from our community revealed that the time interval between collapse and emergency call placement is prolonged and the outcome of cardiac arrest poor in care facilities. A prompt emergency call and cardiopulmonary resuscitation (CPR) after arrest are associated with improved 1-year survival following OHCA. Contrary to accepted wisdom, staff who recognize a cardiac arrest may consult their supervisor and then continue CPR until they receive instructions from him or her.

**Conclusions:**

Manuals or rules for making emergency calls in our public facilities may contain incorrect information, and emergency calls may be delayed owing to correctable human factors. Such manuals should be checked and revised.

## Background

Survival after an out-of-hospital cardiac arrest (OHCA) depends on the following links in the “chain of survival”: immediate recognition of cardiac arrest and activation of the emergency response system, early cardiopulmonary resuscitation (CPR), rapid defibrillation, effective Advanced Life Support, and integrated post-cardiac arrest care [1]. Appropriate and prompt attention to these links, particularly the first three, is essential for survival after OHCA [2-4].

Accumulating evidence shows that a long delay in placing an emergency call worsens the outcome of OHCA [5-7]. Our previous study reported that the delay was most frequently caused by human factors and that long delays were likely to occur in care facilities [5].

In a public facility, an emergency call may occasionally be placed by a member of the staff during business hours. In these cases, though CPR may be initiated early by bystanders, including trained staff, the emergency call may be placed by a staff member according to the institution’s rules and/or manuals.

We hypothesized that the placement of an emergency call might be delayed in care facilities owing to human factors based on an incorrect manual and that a similar problem or a “CPR-first” policy might exist in other facilities in which an emergency call may be made by a staff member or custodian. The purpose of this study was to identify the actions that are prescribed in the rules and/or manuals of public facilities and to determine whether the incidence of delayed emergency call placement and the outcome of OHCA differ among these facilities.

## Methods

Data were collected in accordance with the national ethics guidelines for epidemiological surveys (Ministry of Health, Labor and Welfare in Japan: http://www.mhlw.go.jp/general/seido/kousei/i-kenkyu/index.html). The study was approved by an institutional review board (reference no. 923).

### Populations and settings

The Ishikawa prefecture of Japan encompasses an area of 4,185 km^2^ and has a resident population of 1,160,000. The prefecture is divided into four administrative regions comprising one central or urban region and three semirural or rural regions. Sixty-two percent of the residents are located in the central (urban) region, which has an area of 1,432 km^2^. An estimated 22% of the residents are over the age of 65 years. Population aging is more pronounced in the rural areas than in the urban areas (25.5 vs. 20.3% of residents are over 65 years old, respectively).

There are 11 fire departments and 55 registered ambulances in the Ishikawa prefecture. Each fire department has a dispatch system with telephone-assisted CPR instruction. Emergency medical technicians (EMTs) resuscitate OHCA patients according to a protocol developed by the Ishikawa Medical Control Councils based on the guidelines of the American Heart Association and the Japan Resuscitation Council, unless the patient has post-mortem changes. During resuscitation, paramedics are authorized to perform the following procedures: use of suprapharyngeal airway devices, infusion of Ringer’s lactate through a peripheral vein, and the use of a semi-automated external defibrillator. Since July 2004, specially trained paramedics have been permitted to insert tracheal tubes, and since April 2006 they have been permitted to administer intravenous adrenalin. EMTs are not permitted to terminate resuscitation in the field.

### Questionnaire-based survey regarding emergency calls

The authors collected rules and/or manuals applied when a cardiac arrest or a serious acute illness that could lead to cardiac arrest occurs. The authors sent a questionnaire to public facilities where emergency calls may be occasionally placed by staff members. We identified the care facilities and educational institutions from Ishikawa prefecture websites. A list of municipal public halls/community centers was generated from the homepage of each city. A list of hotels and ryokans (Japanese traditional lodges) was generated from a book of Japanese postal service zip codes, and a list of shopping centers was generated using a telephone number search system. The following types of care facility exist in Japan: residential care facilities for elderly individuals who require short-term daily care; communal daily long-term care for dementia patients; long-term care health facilities; facilities covered by public aid that provide long-term care; and sanatoriums for elderly patients who require long-term care. We excluded residential care facilities from our survey because their capacity is very small and because they are open only in the daytime. We mailed the questionnaire to all of the identified institutions and obtained from each both their reply to the questionnaire and a copy of their manual applied when cardiac arrest and/or serious acute conditions leading to cardiac arrest occur. The questionnaire is presented in Table 1.

**Table 1:** Analysis of the questionnaire survey data

***Questions***	***Ideal response***
Q1. Does your facility have any manual or rule book covering serious medical emergencies in which a person (resident, visitor) becomes unresponsive or his or her physical condition abruptly deteriorates?	Yes
Answer: “Yes” or “No”	
If you answered “Yes” to Q1, please answer Q2 and select one of the items	
If you answered “No” to Q1, please answer Q3 and select one of the items	
Q2. What is the first action that a staff member is required to take for the medical emergency?	Call first immediately
Q3. How should staff members act in the medical emergency?	1. Call 119 first
1. Call 119 first and then examine the victim in detail	
2. First examine the victim in detail and then call 119	
3. Report the situation and then follow the instructions of a supervisor or medical staff member	
4. Immediately initiate CPR or other treatment for the patient and then call 119 if necessary.	
5. Other action (please specify).	
Q4. Does a medical doctor or nurse work every day at your facility? Please choose from the following answers:	1. Works every day
1. Works every day	
2. Works part of the day	
3. Does not work at our facility.	
4. Other (please specify).	
Q5. Does your facility periodically provide basic life support courses?	Yes
Q6. Does your facility have an automated external defibrillator (AED)?	Yes
Q7. Does your facility consult family members regarding what actions to take in the event of a serious medical emergency?	Yes

### Web search for medical emergency manuals in other countries

We conducted a web search using combinations of following terms: “medical”, “emergency”, “procedures”, “manual”, “nursing”, and /or “public facilities”.

### Patient data

Baseline data were prospectively collected for OHCAs that were witnessed or recognized from April 2003 through March 2009 at public facilities in which emergency calls were occasionally made by the staff in accordance with a rule or manual.

The public facilities that were analyzed in this study included various grades of care facility, educational institutions, other facilities at which citizens assemble (such as shopping centers, community centers, sports centers, and recreational facilities), and hotels. The collected data were based on the Utstein template [8,9] and included the region; location of the OHCA; patient’s age, gender, and prior disabilities; any witnesses to the arrest; the etiology of the arrest; the caller; bystander CPR; the identity of the individual who performed CPR; the initial cardiac rhythm; the interval between the recognition of the arrest or the collapse and the call placement; the interval between the call placement and the first CPR; the interval between the call placement and the arrival of EMTs at the patient; and 1-year survival rate. Patients were considered to have survived for 1 year if they were alive in hospital, at home, or in a care and/or rehabilitation facility 1 year after the OHCA. When the emergency call was made after the initiation of CPR or when the interval of the emergency call to the initiation of bystander CPR (the call-bystander CPR interval) was less than 0 min, the reasons for this CPR-first action were identified by the EMTs by interviewing bystanders. The reasons were classified into preventable and unpreventable causes, as described previously [5]. Last, we extracted cases of bystander-witnessed OHCAs in which bystanders performed CPR on their own initiative and used these data to determine differences in the incidence of delayed emergency calls and the outcome of OHCA among public facilities. The primary end point was the survival rate at 1 year. We cleaned all data at least twice before generating the final database by requesting the fire departments to fix the inconsistency in the data.

### Statistical analysis

We analyzed the data using the Joint Medical Program version 8 for Windows (Statistical Analysis System Institute, Cary, NC, USA). Chi-square tests with/without Pearson’s correction or Fisher exact probability tests were applied for univariate analyses. The Kruskal-Wallis test was used for non-parametric comparisons. In all analyses, differences with *p* < 0.05 were considered significant.

## Results

### Analysis of the questionnaire survey data

The response rate to the questionnaire was similar among the different types of public facility and ranged from 45% to 58%. Care facilities and educational institutions were most likely to periodically provide basic life support (BLS) courses (85% and 96%, respectively) and often had manuals that covered serious medical emergencies (89% and 79%, respectively). Eighty-nine percent of all care facility staff were healthcare providers. Automated external defibrillators (AEDs) were installed in every high school and university (100%), but were much less common in care facilities (30.1%). Most of the care facilities (89.2%) had consulted with patients’ family members regarding the actions to take in the event of a serious medical emergency. The most common actions prescribed in a rule or manual to be applied when a cardiac arrest occurred in a care facility or educational institution were to report the situation and then to follow the directions of a supervisor or medical staff member Table 2.

**Table 2:** Characteristics of public facilities and summary of questionnaire survey

	**Care facilities**	**Other facilities**			***p*-value**
		**High schools and universities**	**Community centers**	**Hotels and shopping centers**	
Responses to the questionnaire, *n*/mailed number (%)	157/269 (58.4)	47/83 (56.6)	229/487 (47.0)		0.2111
			120/245 (49.0)	109/242 (45.0)	0.3373
Manual or rule for serious medical emergencies, *n*/total responses (%)	138/155 (89.0)	37/47 (78.7)	56/228 (24.6)		<0.0001
			11/121 (9.1)	45/107 (42.1)	<0.0001
Healthcare provider, *n*/total responses (%)					
Works every day	101/153 (66.0)	19/46 (41.3)	7/225 (3.1)		<0.0001
			2/120 (1.7)	5/105 (4.8)	<0.0001
Works part of the day	35/153 (22.9)	3/46 (6.5)	2/225 (0.9)		<0.0001
			1/120 (0.8)	1/105 (1.0)	<0.0001
None	17/153 (11.1)	24/46 (52.2)	216/225 (96.0)		<0.0001
			117/120 (97.6)	99/105 (94.3)	<0.0001
Provide Basic Life Support course periodically, *n*/total responses (%)	129/152 (84.9)	44/46 (95.7)	107/224 (47.8)		<0.0001
			64/119 (53.8)	43/105 (41.0)	<0.0001
Automated external defibrillator (AED) installed, *n*/total responses (%)	46/153 (30.1)	46/46 (100)	91/225 (40.4)		0.0394
			48/120 (40.0)	43/105 (41.0)	<0.0001
Consult family members regarding what actions to take in the event of a serious medical emergency, *n*/total responses (%)	132/148 (89.2)	–	–	–	
Actions prescribed in a manual and applied when cardiac arrest occurs (%)					
Call 119 first and then check the victim in detail	43/155 (27.7)	17/45 (37.8)	170/227 (74.9)		<0.0001
			96/120 (80.0)	74/107 (69.2)	<0.0001
Call 119 after checking the victim in detail	14/155 (9.0)	7/45 (15.6)	27/227 (7.1)		<0.0001
			14/120 (11.7)	13/107 (12.1)	<0.0001
Report the situation and follow instructions from a supervisor or medical staff	86/155 (55.5)	16/45 (35.6)	12/227 (5.3)		<0.0001
			3/120 (2.5)	9/107 (8.4)	<0.0001
Start CPR or other treatment immediately and call 119 if necessary	12/155 (7.7)	5/45 (11.1)	18/227 (7.9)		<0.0001
			7/120 (5.8)	11/107 (10.3)	<0.0001

### Web search results

We identified 16 full text manuals from three public institutes in the US and 5 universities in the US, Canada, Austria and the UK. Thirteen manuals prescribed “call first” (81%); “other actions” included contacting a trained first aid officer, reception, facility manager, administration, or security officer.

### Characteristics and outcomes of OHCA patients

As shown in Table 3, female patients, older patients, prior disabilities, and OHCAs of non-cardiac etiology were significantly more common in care facilities. Both the interval between arrest recognition/collapse and placement of the emergency call and the interval between emergency call placement and the arrival of EMTs were longer in care facilities. In care facilities, the incidence of CPR before EMT arrival was higher, and CPR was more frequently initiated before the emergency call. Resuscitation was most frequently attempted by healthcare providers at the care facilities, and the emergency call was most frequently placed by a staff member (99%, compared with 86% for educational institutions and 56% for other institutions, *p* < 0.0001). Concerning the incidence of CPR-first cases with a subtractive call – bystander CPR interval value was 53.9% in care facilities and 49.2% in other facilities for OHCAs with bystander CPR. In most of the CPR-first cases (98.8% in care facilities and 60.6% in other facilities), the cause of the delayed emergency call was preventable.

**Table 3:** Differences between care facilities and other facilities in characteristics of OHCA patients

	**Care facilities**	**High schools and universities**	**Others**	***p*-value**	**Odds ratio (95% CI)**
	**(*n *= 556)**	**(*n *= 8)**	**(*n *= 386)**		
		**Other public facilities**			
		**(*n *= 394)**			
Region	262 (47.1)	7 (87.5)	192 (49.7)	0.4290*	0.900 (0.694–1.168)*
Central or urban (%)		199 (50.5)		0.3037	0.873 (0.674–1.131)
Patient’s gender	197 (35.4)	6 (75.0)	299 (77.5)	<0.0001*	0.160 (0.119–0.215)*
Male (%)		305 (77.4)		<0.0001	0.160 (0.119–0.215)
Patient’s age, years, median (25–75%)	86 (81–91)	17 (16–64)	68 (56–78)	<0.0001*	Undefined
		68 (55–78)		<0.0001	Undefined
Patient’s prior disabilities	137 (24.6)	8 (100)	318 (82.4)	<0.0001*	0.070 (0.051–0.097)*
None (%)		326 (82.7)		<0.0001	0.068 (0.049–0.094)
Etiology	280 (50.4)	3 (37.5)	222 (57.5)	0.0303*	0.749 (0.577–0.973)*
Cardiac (%)		225 (57.1)		0.0399	0.762 (0.588–0.988)
Arrest	324 (58.3)	2 (25.0)	217 (55.1)	0.5304*	1.088 (0.837–1.414)*
Witnessed (%)		219 (55.6)		0.4093	1.116 (0.860–1.448)
Interval from arrest recognition/collapse to call, min, median (10-25-75-90%)	2	2.5	2	0.0006*	Undefined
		(0-1-7-9)	(0-1-4-8)		
	(−1-1-6-13)	2 (0-1-4-8)		0.0007	Undefined
CPR before EMT arrival (%)	455 (81.8)	5 (62.5)	182 (47.2)	<0.0001*	5.050 (3.763–6.775)*
		187 (47.5)		<0.0001	4.987 (3.712–6.681)
CPR performer	339/455 (74.5)	0/5 (0)	33/182 (18.1)	<0.0001*	12.719 (8.293–19.505)*
Healthcare provider, *n*/total BCPR (%)		33/193 (17.1)		<0.0001	13.208 (8.622–20.234)
CPR first, *n*/total BCPR (%)	245/455 (53.9)	3/5 (60.0)	89/182 (48.9)	0.4994	1.219 (0.864–1.720)*
		92/187 (49.2)		0.2839	1.205 (0.857–1.694)
CPR first for preventable reason, *n*/total CPR first (%)	80/81 (98.8)	0/2 (0)	20/31 (64.5)	<0.0001	44 (5.362–361.091)*
		20/33 (60.6)		<0.0001	52 (6.418–421.316)
Interval from call to arrival of EMT at patient, min, median (25–75%)	8 (6–11)	5 (5–6.5)	7 (5–9)	<0.0001*	Undefined
		7 (5–9)		<0.0001	Undefined
Emergency call placed by institution staff, *n*/total (%)	538/542 (99.3)	6/7 (85.7)	91/164 (55.5%)	<0.0001*	107.9 (38.5–302.4)*
		97/171 (56.7)		<0.0001	102.6 (36.7–287.2)

*CI* confidence interval, *CPR* cardiopulmonary resuscitation, *EMT* emergency medical technician, *BCPR* bystander cardiopulmonary resuscitation.

*Comparison between care facilities and other facilities excluding schools.

**Figure 1:**
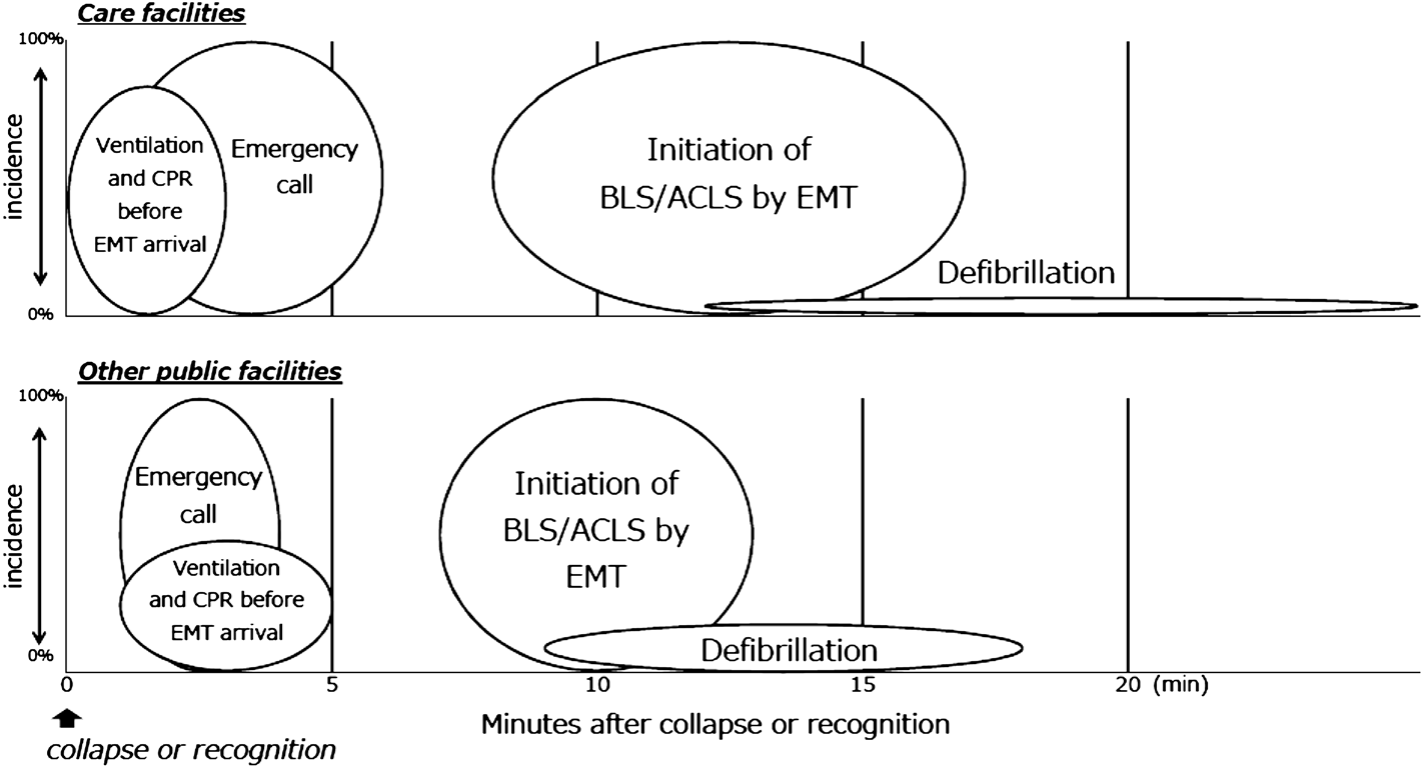
**Placements of links in the "chain of survival" for care facilities and other facilities.** The *widths of circles* represent an interquartile range (25-75%). The *heights of circles* denote the incidence. *CPR* cardiopulmonary resuscitation, *EMT* emergency medical technician, *BLS* basic life support, *ACLS* advanced cardiovascular life support.

**Figure 2:**
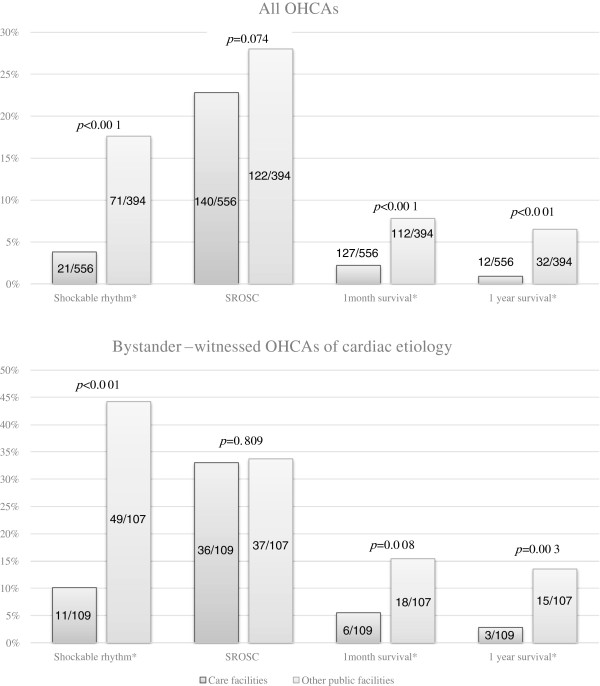
**Comparison of the outcome and incidence of VF/VT in relation to location.***SROSC* sustained return of spontaneous circulation. *Significantly different between care facilities and other public facilities (*p* < 0.05).

The links in the chain of survival for care and other facilities are illustrated in Figure 1. The median interval between arrest recognition/collapse and the emergency call was 2 min [interquartile range (IQR) = 1–6] in care facilities and 2 min (IQR = 1–4) in other facilities (*p* < 0.0001). The median interval between arrest recognition/collapse and the initiation of CPR before EMT arrival was 1 min (IQR = 0–3) in care facilities and 2 min (IQR = 1–5) in other facilities (*p* = 0.0007). The median interval between arrest recognition/collapse and the initiation of resuscitation by EMTs was 8 min (IQR = 6–11) in care facilities and 7 min (IQR = 5–9) in other facilities (*p* < 0.0001). The median interval between arrest recognition/collapse and defibrillation was 18 min (IQR = 12–26) in care facilities and 12 min (IQR = 9–18) in other facilities (*p* = 0.0336). As shown Figure 2, the incidence of ventricular fibrillation/ventricular tachycardia as the initial rhythm and survival rates at 1 month and 1 year were significantly lower in care facilities.

### Survival at 1 year in six groups of bystander-witnessed OHCAs with bystander-initiated CPR

We extracted 192 cases of bystander-witnessed OHCAs in which bystanders performed CPR on their own initiative. The median collapse-call and call-CPR intervals were 2 min (IQR = 1–5) and 1 min (IQR = 0–3), respectively. We classified the 192 cases into six groups according to the collapse-call and call-CPR interval. As shown in Table 4, there were no survivors at 1 year when the emergency call was considerably delayed (collapse-call interval ≥6 min). When the emergency call was not considerably delayed (collapse-call interval <6 min), survival at 1 year was greatest in cases without a long delay in CPR (cases with a call-CPR interval <3 min). The 1-year survival rate appeared to be lower in 54 CPR-first cases (call-CPR interval <0 min) than in 94 cases in which CPR was undertaken in the standard order (call first: call-CPR interval = 0–3 min). However, this difference was not statistically significant (*p* = 0.487, Fisher’s exact probability test).

**Table 4:** Survival at 1 year in six groups of bystander-witnessed OHCAs where bystanders performed CPR on their own initiative

**Category**	**Interval from call to bystander CPR**	**Considerable delay of emergency call**	
		**No**	**Yes**
**Call first or CPR first**			
		**(interval from collapse to call <6 min)**	**(interval from collapse to call ≥6 min)**
Call first with considerable delay of CPR	>3 min, *n*/total	0/5 (0%)	0/20 (0%)
Standard call first with small delay of CPR	0–3 min, *n*/total	7/94 (6.6%)	0/10 (0%)
CPR first	<0 min, *n*/total	2/54 (1.9%)	0/9 (0%)

Difference in 1-year survival between standard call first and CPR first groups (shadowed boxes) was not statistically significant (*p* = 0.487, Fisher’s exact probability test).

## Discussion

Our questionnaire survey disclosed that emergency manuals for serious injury and illness in Japanese public facilities frequently give priority to reporting the incident to a custodian or supervisor rather than to making an immediate emergency call or notifying emergency medical services (EMSs). Incorrect manuals are most frequently found in care facilities and educational institutes. The incidence of emergency calls made after instructions from a custodian or supervisor was high in facilities where the call was placed by a member of staff; it was highest in care facilities and lowest in public facilities other than educational institutions. Staff who witness or recognize a cardiac arrest may consult their supervisor first and then perform CPR until they receive instructions from him or her. This delay before an emergency call is made is presumably related to the low survival rate of OHCAs in care facilities.

Japanese care facilities are multifarious. Many elderly people with minor disabilities live in facilities for daily life care, owing to the aging population and the increasing number of nuclear families comprising entirely aged members [10]. In care facilities providing medical care, members of the family of patients with serious disabilities are consulted regarding the actions to take in the event of a medical emergency. Not all patients who suffer a cardiac arrest in these facilities are transported by the EMSs. We found that BLS courses were periodically held in most of the care facilities and resuscitation was started early. Nevertheless, the outcome of cardiac arrest at care facilities was poor.

Our questionnaire-based survey revealed differences in AED availability among public facilities. Care facilities were the least likely to have an AED installed (30%), and high schools and universities were most likely (100%). This suggests that the delay of emergency calls in care facilities might be associated with a delay of AED use by EMTs and the low incidence of shockable initial rhythm.

Our web search disclosed that most manuals in colleges in other countries prescribed call-first actions [11-14]. The manuals state simply that staff should make an emergency call immediately after they encounter a serious illness or medical emergency. However, we failed to find any manuals applied in care facilities in our web search. A further questionnaire survey may be necessary to determine whether incorrect manuals are present in care facilities internationally.

Incorrect manuals that upset the order of the links in the chain of survival were present not only in care facilities but also in schools. However, we were unable to perform a precise comparison of the backgrounds and outcomes of OHCAs between educational and other facilities because the number of OHCAs that occurred at educational facilities was low. Further investigation will be necessary to determine BLS actions at schools and universities.

Previous studies in the US have reported that OHCA patients in care facilities are frequently elderly males with a disability or illness and that the incidence of witnessed cardiac arrest ranges from 38% to 49% [15,16]. It has also been reported that the incidence of bystander CPR is higher in care facilities than in other public facilities [17]. The characteristics and backgrounds of the OHCAs in the present study are consistent with those in previous reports. Although the quality of bystander CPR was not determined in our study, periodic BLS courses were held in most of the care facilities. The quality of bystander CPR has been reported to be high in locations where cardiac arrests occur frequently [18]. Our study showed that bystander CPR in care facilities is predominantly performed by a trained staff member. The emergency call was most frequently placed by a staff member, and the interval between arrest recognition and placement of the emergency call was significantly prolonged in care facilities compared with other public facilities. A delay in this critical interval in care facilities has not been reported in communities other than ours.

In agreement with previous reports [19,20], this study showed that there is a high incidence of non-shockable initial rhythm and a low survival rate among OHCAs in care facilities. Shah et al. proposed that a low incidence of witnesses to OHCA and a high incidence of pre-existing complications are the main reasons for this low survival rate [16]. In the present study, univariate analysis revealed that the incidence of prior disability was higher in care facilities than in other public facilities. The likelihood of a return of spontaneous circulation and survival after an OHCA decreases with age [21,22], and we found the age of OHCA patients to be much higher in care facilities. Previous studies have reported incidences of witnessed OHCAs of 38–44% [16,23]. In our region, the incidence of witnessed OHCAs at care facilities (58.3%) was as high as that at other public facilities (55.6%). The low survival rate of OHCAs in care facilities may therefore be attributed to a combination of multiple factors that are related to the setting, including delays in placing emergency calls.

### Limitations

The response rate to our questionnaire-based survey was lower than we had expected, the prospective cohort study and the questionnaire survey were not linked, and the results of the questionnaire did not necessarily reflect the circumstances at the public facilities that were analyzed in the prospective cohort study. However, it is clear that intervention, including education of facility staff and revision of manuals, is necessary to resolve this common issue in public institutions, particularly care facilities, high schools, and universities.

## Conclusions

Our questionnaire-based survey showed that emergency manuals for dealing with serious injury or illness in Japanese public facilities give priority to reporting the incident to a custodian or supervisor rather than to making an immediate emergency call or notifying EMSs. Such incorrect manuals are common in care facilities and educational institutes, and emergency manuals in all public facilities should be checked and revised.

## Abbreviations

OHCA: Out-of-hospital cardiac arrest; CPR: Cardiopulmonary resuscitation; BLS: Basic life support; EMT: Emergency medical technicians; SROSC: Sustained return of spontaneous circulation; AED: Automated external defibrillator.

## Competing interests

The authors state that we have no competing interests.

## Authors’ contributions

YT was coordinated the manuscript submissions. He conducted background research, performed data analysis, and drafted the manuscript. TN helped to design the survey and assisted with data analysis. He contributed substantially to the manuscript revision. KT and TK assisted with data analysis. HI was the country coordinator who oversaw data collection. He contributed substantially to data interpretation and to the manuscript writing. HI takes responsibility for the paper as a whole. All authors read and approved the final manuscript.
